# Associations between parental history of dementia and plasma markers of inflammation in a multi‐ethnic middle‐aged community of adults

**DOI:** 10.1002/alz.71355

**Published:** 2026-04-12

**Authors:** Edric D. Winford, Benjamin D. Huber, Dominika Seblova, Jeffrey Pyne, Justina F. Avila‐Rieger, Indira C. Turney, Jessica A. Mazen, Adam M. Brickman, Jennifer J. Manly

**Affiliations:** ^1^ Department of Neurology Taub Institute for Research on Alzheimer's Disease and the Aging Brain Gertrude H. Sergievsky Center Vagelos College of Physicians and Surgeons Columbia University New York New York USA; ^2^ Department of Public Health Second Faculty of Medicine Charles University Prague Czech Republic; ^3^ Laboratory of Epidemiology and Population Sciences National Institute on Aging National Institutes of Health Baltimore Maryland USA

**Keywords:** Alzheimer's disease, dementia, parental history, peripheral inflammation

## Abstract

**INTRODUCTION:**

Parental history of dementia is associated with increased dementia risk. We investigated whether having a parent with dementia is associated with increased peripheral inflammation in middle‐aged adults.

**METHODS:**

Participants were from the Offspring Study (*n* = 1204). Parental dementia status was determined by a diagnostic consensus conference. Plasma chemokine and cytokine concentrations were assayed with Luminex technology.

**RESULTS:**

Parental history of dementia was associated with higher levels of eotaxin and lower levels of granulocyte colony‐stimulating factor, vascular endothelial growth factor A, and interleukin (IL)‐27. IL‐18 and epidermal growth factor levels were higher in Black individuals with a parental history of dementia compared to Hispanic individuals with the same history. Women with a parental history of dementia had higher levels of interferon‐alpha 2, IL‐12p70, soluble CD40 ligand, and IL‐18 compared to men with the same history.

**DISCUSSION:**

Parental history of dementia is associated with elevated markers of peripheral inflammation. These associations vary across sex, race, and ethnicity.

## Highlights

- Parental dementia history was linked to elevated pro‐inflammatory cytokines in midlife.
- Age‐related increases in inflammation were stronger with parental dementia history.
- Peripheral inflammation in parental dementia history differed by age, sex, and ethnicity.

## 1 BACKGROUND

Peripheral inflammation has emerged as a contributing factor to the development of Alzheimer's disease (AD).^1^ People with AD have increased plasma inflammatory cytokine levels, such as interleukin (IL)‐1β, IL‐6, interferon gamma (IFN‐γ), and C‐reactive protein (CRP), compared to cognitively unimpaired individuals.^2^, ^3^ Elevated peripheral cytokine concentrations are also associated with an increased risk of incident mild cognitive impairment (MCI) and dementia in cognitively unimpaired individuals.^4^, ^5^, ^6^, ^7^ However, higher levels of some peripheral cytokines, like IFN‐γ and IL‐12p70, are associated with slower cognitive decline,^8^ suggesting both risk and protective inflammatory effects.

Individuals with a first‐degree family history of AD have a 4 to 10 times greater risk of developing AD,^9^, ^10^, ^11^ higher levels of amyloid beta (Aβ) on positron emission tomography (PET),^12^ and lower cognitive scores^13^ than those without such history. Peripheral inflammation may contribute to these differences, as animal studies in AD demonstrated that peripheral inflammation can reduce microglia's ability to clear Aβ^14^ and in young mice, peripheral inflammation can lead to reduced neurogenesis and memory.^15^ In a longitudinal study of cognitively unimpaired middle‐aged adults with a parental history of AD, soluble platelet‐derived growth factor receptor β (sPDGFRβ), a marker of vascular integrity, was positively associated with cerebrospinal fluid (CSF) AD biomarkers including tau and Aβ40, as well as inflammatory markers such as IL‐9 and tumor necrosis factor (TNF) receptors, which are key mediators of inflammation.^16^ However, these individuals were not compared to people without a parental history of AD. These studies suggest a potential mechanism of intergenerational transmission of risk for AD via peripheral inflammation.

Black and Hispanic individuals have an increased incidence and prevalence of AD compared to non‐Hispanic White people.^17^, ^18^ Minoritized populations have increased peripheral inflammation, indexed by cytokines, including IL‐6 and CRP,^19^, ^20^, ^21^ compared to non‐Hispanic White people, likely due to inequalities in socioeconomic status (SES) and psychosocial stressors.^22^ Women also have an increased risk for AD, accounting for two thirds of cases, and have a faster disease progression once diagnosed.^23^ Peripheral inflammation, which itself differs by sex, may contribute to these disparities. Several pro‐inflammatory cytokines (IL‐6, IL‐1β, and TNF‐α) implicated in AD^24^ are regulated by the female hormone estrogen, which may influence inflammatory responses in women. Inflammatory markers are also associated with sex‐specific patterns in memory decline.^25^, ^26^ These findings highlight the importance of examining how inflammatory pathways in AD may vary across sex, race, and ethnicity.

In the current study, we examined markers of peripheral inflammation in the Offspring Study, a well‐characterized, community‐based study cohort of aging and cognition, to test the hypothesis that individuals with a parental history of dementia have increased levels of peripheral inflammation compared to individuals without a parental history of dementia across age, race and ethnicity, and sex. We also wanted to understand the relationship between these inflammatory markers and cognition.

## 2 METHODS

### 2.1 Participants

The participants came from the Offspring Study, a community‐based longitudinal study of aging and cognition designed to identify the social, structural, and biological pathways of racial and ethnic disparities in AD. Offspring Study participants are the adult children of participants in the Washington Heights Inwood Columbia Aging Project (WHICAP), a multiethnic, community‐based longitudinal study of aging in dementia in northern Manhattan consisting of eligible adults ≥ 65 years.^23^, ^24^ Offspring study participants are ≥ 28 years of age, fluent in English or Spanish, and willing to donate blood, and were evaluated between January 2017 and March 2020. Participants completed informed consent forms, and all study protocols were approved by the Columbia University Institutional Review Board.

### 2.2 Parental dementia status

Parental history of dementia was determined through diagnostic consensus conferences conducted as part of WHICAP. At each visit, participants underwent assessments including physical, neurological, and neuropsychological testing, and blood collection. Clinical diagnoses of dementia, including probable or possible AD and related dementias, were assigned according to the National Institute on Aging–Alzheimer's Association criteria, based on cognitive testing and functional assessments.^27^ All diagnostic decisions were made by a consensus panel including at least one neurologist and one neuropsychologist, blinded to previous diagnoses during follow‐up. The distribution of parental dementia diagnoses is provided in Table S1 in supporting information.

### 2.3 Cytokine quantification

Blood samples were collected into Becton Dickinson (BD) Vacutainer tubes containing ethylenediaminetetraacetic acid (EDTA). Samples were centrifuged at 2000 × g for 15 minutes at room temperature. The resulting plasma was aliquoted into polypropylene cryotubes and stored at −80°C until analysis. Cytokine concentrations were quantified with the Luminex MILLIPLEX MAP Human Cytokine/Chemokine Magnetic Bead Panel (HCYTOMAG‐60K) and the High Sensitivity T Cell Panel (HSTCMAG‐28SK) according to the manufacturer's protocols. CRP levels were measured separately using Integra technology.^28^ Cytokine measurements with a coefficient of variation > 20% were not included. For cytokine measurements below the level of detection, we imputed the measurement value as the lower detection limit value divided by the square root of 2, which is consistent with the midpoint between 0 and the detection limit for a log‐normal distribution.^29^ Cytokines with > 60% of measurements below the level of detection were excluded from the analysis.^28^ We retained 42 cytokines for analysis: IL‐1α, IL‐1β, IL‐1 receptor antagonist (IL‐1RA), IL‐4, IL‐5, IL‐6, IL‐8, IL‐9, IL‐12p40, IL‐12p70, IL‐13, IL‐15, IL‐17A, IL‐17E/IL‐25, IL‐18, IL‐27, interferon alpha 2 (IFN‐α2), IFN‐γ, TNF‐α and TNF‐β, soluble CD40 ligand (sCD40L), high‐sensitivity CRP (hsCRP), eotaxin‐1 (CCL11), monokine induced by gamma interferon (MIG/CXCL9), macrophage inflammatory proteins (MIP‐1α/CCL3 and MIP‐1β/CCL4), monocyte chemoattractant proteins (MCP‐1/CCL2 and MCP‐3/CCL7), macrophage‐derived chemokine (MDC/CCL22), interferon gamma‐induced protein 10 (IP‐10/CXCL10), regulated upon activation normal T cell expressed and secreted (RANTES/CCL5), and fractalkine (CX3CL1), granulocyte colony‐stimulating factor (G‐CSF), granulocyte‐macrophage colony‐stimulating factor (GM‐CSF), macrophage colony‐stimulating factor (M‐CSF), fms‐like tyrosine kinase 3 ligand (Flt‐3L), vascular endothelial growth factor A (VEGF‐A), epidermal growth factor (EGF), platelet‐derived growth factors (PDGF‐AA and PDGF‐BB), fibroblast growth factor 2 (FGF‐2), and transforming growth factor alpha (TGF‐α). All cytokine concentration values were log‐transformed.

### 2.4 Neuropsychological assessment

Participants completed a neuropsychological battery in either English or Spanish at the same time as blood collection. A general cognition score was derived with a confirmatory factor analysis based on pre‐specified hypotheses for factor structure. The general cognition factor was specified as a second‐order factor with five cognitive domain indicators—executive function/working memory, memory, language, speed, and visuospatial—as indicators.

Indicators for the executive function/working memory factor included National Institutes of Health (NIH) Toolbox scores on List Sorting Working Memory, Dimensional Change Card Sort, and Flanker Inhibitory Control and Attention, and Digit Span, with method correlations between Digit Span forward and backward, and Card Sort and Flanker. The language factor included Category Fluency (animals and vegetables), Letter Fluency (CFL in English, PMR in Spanish), and the Multilingual Naming Test (MINT), with method correlations between animal and vegetable fluency. Indicators for the speed domain included the Pattern Comparison Processing Speed test from the NIH Toolbox, Wechsler Adult Intelligence Scale Third Edition Digit Symbol, and the Color Trails Test, with a method correlation between the forward and backward trails test scores. Memory domain indicators were Immediate and Delayed Recall from the Craft story and immediate, delayed free recall, and delayed recognition scores from the Selective Reminding Test (SRT), with method correlations among the Craft items and the SRT items. The visuospatial domain indicators included the Copy and Delayed Recall scores for the Benson Complex Figure test.

**RESEARCH In CONTEXT**

1. **Systematic review**: PubMed and Google Scholar databases were searched to identify scientific articles on parental dementia history in relation to inflammation, sex, race, ethnicity, and cognition.
2. **Interpretation**: Individuals with a parental history of dementia showed early signs of pro‐inflammatory peripheral immune alterations in midlife. These alterations were influenced by age, sex, and race/ethnicity.
3. **Future directions**: To the best of our knowledge, this study is the first to examine associations between peripheral inflammation and parental history of dementia in midlife. Future studies are necessary to clarify the mechanisms linking parental history of dementia to increased inflammation. Additionally, future studies should also examine the immune cell landscape in individuals with a parental history of dementia to determine specific shifts in immune composition that may contribute to early peripheral inflammation.

Scores were estimated with full information maximum likelihood to allow for score estimation even with missing values. Calculations were completed with lavaan version 0.6‐19, and R version 4.1.1. Cognition scores were converted to *Z* scores based on the mean and standard deviation from the baseline administration of the measures in the Offspring cohort.

### 2.5 Psychological assessment

Given that psychological factors such as stress and depression have been linked with peripheral inflammation,^30^, ^31^, ^32^ we evaluated whether these factors differed by parental dementia status to determine whether group differences in inflammation might reflect differences in psychological symptoms. We used anxiety and depression as outcomes. Anxiety was assessed using a 5‐item subset of the Beck Anxiety Inventory,^33^, ^34^ evaluating five dimensions of anxiety experienced during the previous week. Responses ranged from 0 (never) to 3 (most of the time). Items were summed to create a total score ranging from 0 to 15, with higher scores indicating greater anxiety. Depressive symptoms were measured using the 10‐item short form of the Center for Epidemiological Studies Depression Scale (CES‐D‐10),^35^ which evaluates 10 symptoms of depression experienced during the previous week. After reverse‐coding positive items, scores were summed to create a total ranging from 0 to 30, with higher scores indicating more depressive symptoms. Only participants who completed the full questionnaire for both anxiety and depression were included.

### 2.6 Covariates/moderators

Initial analyses included age and sex as covariates when appropriate. We also added terms for the interaction of parental history of dementia with race and ethnicity and apolipoprotein E (*APOE*) genotype. Race and ethnicity were self‐reported. Main analyses included all participants. However, because the vast majority of participants in the study identified as Black, White, or Hispanic, we excluded 14 participants who identified as Asian, American Indian, multiracial, or did not specify race and ethnicity in models with race and ethnicity interactions. In all analyses, Hispanic was used as the reference group for race and ethnicity, and female was used as the reference group for sex. For *APOE* ε4 interactions, participants were grouped by having one or two ε4 alleles versus no ε4 alleles.

### 2.7 Statistical analysis

Unadjusted associations between cytokine concentrations and parental history of dementia status were estimated using bivariable linear regressions, with log‐transformed cytokine concentrations as the outcome. Additional linear regression models further adjusted associations between cytokines and parental history of dementia status by age and sex. We reported both raw and Benjamini–Hochberg multiple comparison adjusted *P* values, with a false discovery rate controlled at 5%. We tested for interactions between parental dementia risk status and age, race and ethnicity, sex/gender, and *APOE* ε4 status by adding the interaction terms to the adjusted models separately. Associations between cytokines and cognitive factors were tested with bivariable linear models and multivariable models adjusting for sex and age. All *P* values were adjusted for multiple comparisons across all cytokines using Benjamini–Hochberg with a 5% false discovery rate.

## 3 RESULTS

### 3.1 Demographic characteristics

Table 1 describes the sample of 1204 adults, including 851 Black individuals (21%), 249 Hispanic individuals (70%), 90 White individuals (7%); individuals who identified as American Indian (*N* = 1), multiracial (*N* = 5), or did not specify race or ethnicity (*N* = 8) were combined into the other category (*N* = 14; 1%). There were 808 women (67%). Hispanic participants were from the Dominican Republic (734), Puerto Rico (45), Cuba (30), Ecuador (29), and other countries (13). The study was separated into two groups: individuals without a parental history of dementia (*n* = 778) and individuals with a parental history of dementia (*n* = 426). Individuals with a parental history of dementia were older on average, while individuals without a parental history of dementia were more likely to be women, English speaking, *APOE* ε4 carriers, and have higher education.

**TABLE 1:** Baseline characteristics of the study sample.

Offspring Sample	Total	No parental dementia	Parental dementia
Characteristic	(*N* = 1204)	(*N* = 778)	(*N* = 426)
Age, mean (SD)	56.0 (11.2), 28.0–89.0	54.0 (10.9), 28.0–83.0	59.7 (10.8), 29.0–89.0
**Sex/gender, *N* (%)**			
Female	808 (67%)	506 (65%)	302 (71%)
Not female	396 (33%)	272 (35%)	124 (29%)
**Race and ethnicity, *N* (%)**			
Hispanic	851 (71%)	483 (62%)	368 (86%)
Non‐Hispanic Black	249 (21%)	202 (26%)	47 (11%)
Non‐Hispanic White	90 (7.5%)	83 (11%)	7 (1.6%)
Other	14 (1.2%)	10 (1.3%)	4 (0.9%)
**Primary language, *N* (%)**			
English	626 (52%)	498 (64%)	128 (30%)
Spanish	578 (48%)	280 (36%)	298 (70%)
Years of education, mean (SD)	12.8 (3.8), 0.0–20.0	13.4 (3.5), 0.0–20.0	11.6 (4.1), 0.0–20.0
Unknown	46	24	22
*APOE* ε4 status, *N* (%)	(N = 998)	(N = 629)	(N = 369)
ε4 carrier	710 (71%)	465 (74%)	245 (66%)
Non‐ε4 carrier	288 (29%)	164 (26%)	124 (34%)
Unknown	206	149	57

*Notes*: Mean (SD), Min‐Max; *n* / *N* (%).

Abbreviations: *APOE*, apolipoprotein E; SD, standard deviation.

### 3.2 Associations of parental history of dementia with peripheral cytokines

**FIGURE 1:**
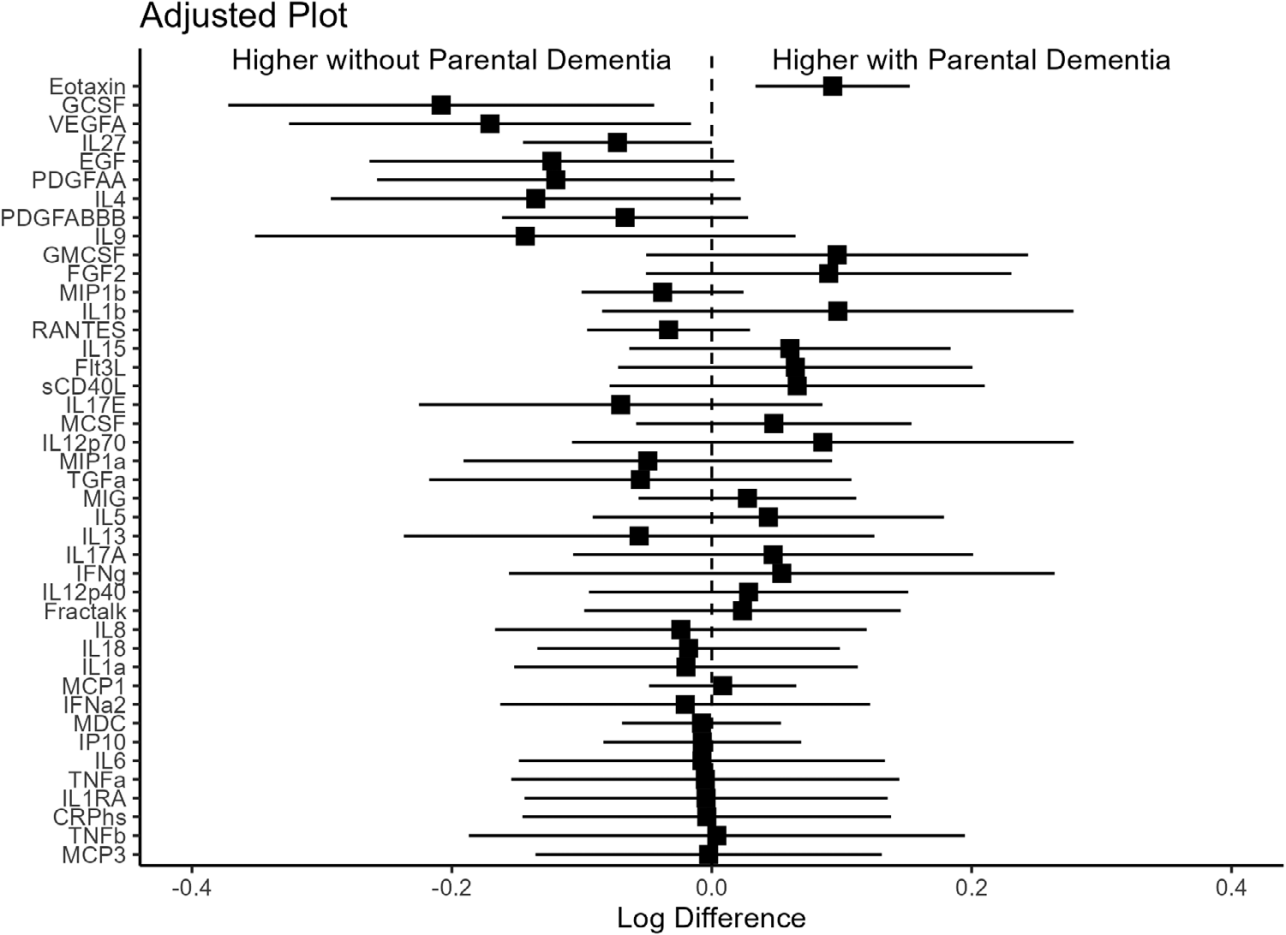
Forest plots of adjusted linear regression models examining the associations between peripheral cytokines and chemokines and parental history of dementia. Beta coefficients reflect the difference in log‐transformed cytokine concentrations between individuals with and without a parental history of dementia. Error bars represent 95% confidence intervals.

In the full sample, unadjusted models showed that eotaxin (log‐diff: 0.13, 95% confidence interval [CI]: 0.07, 0.19) and MIG (0.17, 95% CI: 0.08, 0.25) were higher in individuals with a parental history of dementia, while G‐CSF (−0.25, 95% CI: −0.4, −0.25), IL‐9 (−0.26, 95% CI: −0.46, −0.06), EGF (−0.17, 95% CI: −0.31, −0.04), VEGF‐A (−0.19, 95% CI: −0.34, −0.04), and IL‐4 (−0.18, 95% CI: −0.33, −0.02) were lower in individuals with a parental history of dementia (Figure S1 in supporting information). After adjusting for covariates, eotaxin (0.09, 95% CI: 0.03, 0.15) remained significantly increased. G‐CSF (−0.21, 95% CI: −0.38, 0.05), VEGF‐A (−0.17, 95% CI: −0.32, 0.01), and IL‐27 (−0.07, 95% CI: −0.15, 0) were lower in individuals without a parental history of dementia than those with a parental history of dementia. However, none of these differences remained significant after correction for multiple comparisons (Figure 1).

### 3.3 Interactions between parental history of dementia and age on peripheral cytokines

**FIGURE 2:**
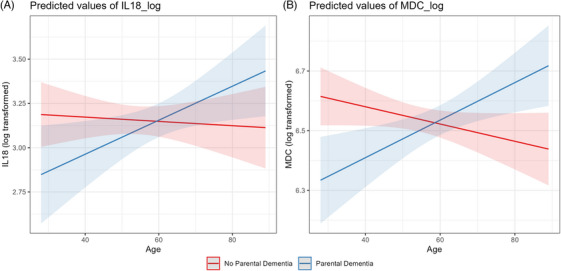
Interaction between age and parental history of dementia on log‐transformed (A) IL‐18 and (B) MDC levels. Shaded areas represent 95% confidence intervals. Individuals with a parental history of dementia (blue) and individuals without parental dementia history (red). IL, interleukin; MDC, macrophage‐derived chemokine.

Because several cytokines increase with age,^36^ we wanted to determine whether age‐related changes in peripheral inflammation differ by parental history of dementia. To test this possibility, we examined interactions between age and parental dementia status on cytokine levels. The positive association of age with IL‐18 concentrations (β_int_ = 0.01, 95% CI: 0.00, 0.02, *P* = 0.034; Figure 2A) and MDC concentrations (β_int_ = 0.00, 95% CI: 0.00, 0.01, *P* ≤ 0.001; Figure 2B) was stronger among individuals with a parental history of dementia.

### 3.4 Interactions between parental history of dementia and race/ethnicity on peripheral cytokines

**FIGURE 3:**
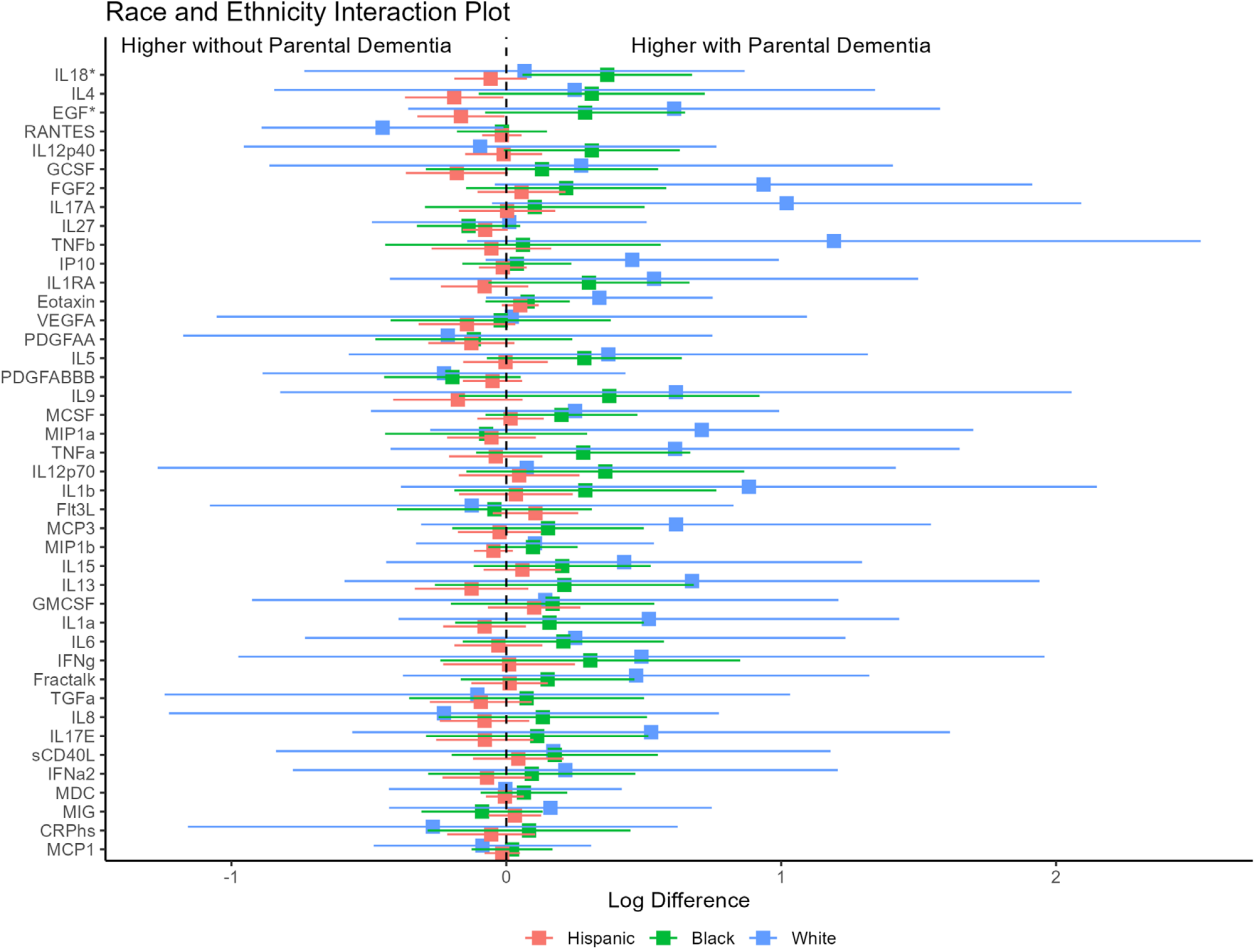
Forest plots of linear regression models examining the interactions between parental history of dementia and race/ethnicity on peripheral cytokines. Beta coefficients and 95% confidence intervals are shown separately for Hispanic (red), Black (green), and White (blue) participants. Asterisks (*) indicate cytokines with a significant interaction effect (*p*  <  0.05) between parental history and race/ethnicity.

The association between parental history of dementia and EGF levels was greater among Black participants than Hispanic participants (β for parental dementia history x race and ethnicity interaction = 0.46, 95% CI: 0.07–0.85, *P* = 0.02). Similarly, the association between parental history of dementia and IL‐18 levels was greater among Black participants compared to Hispanic participants (β_int_ = 0.42, 95% CI: 0.09–0.75, *P* = 0.01; Figure 3).

### 3.5 Interactions between parental AD history and sex/gender on peripheral cytokines

**FIGURE 4:**
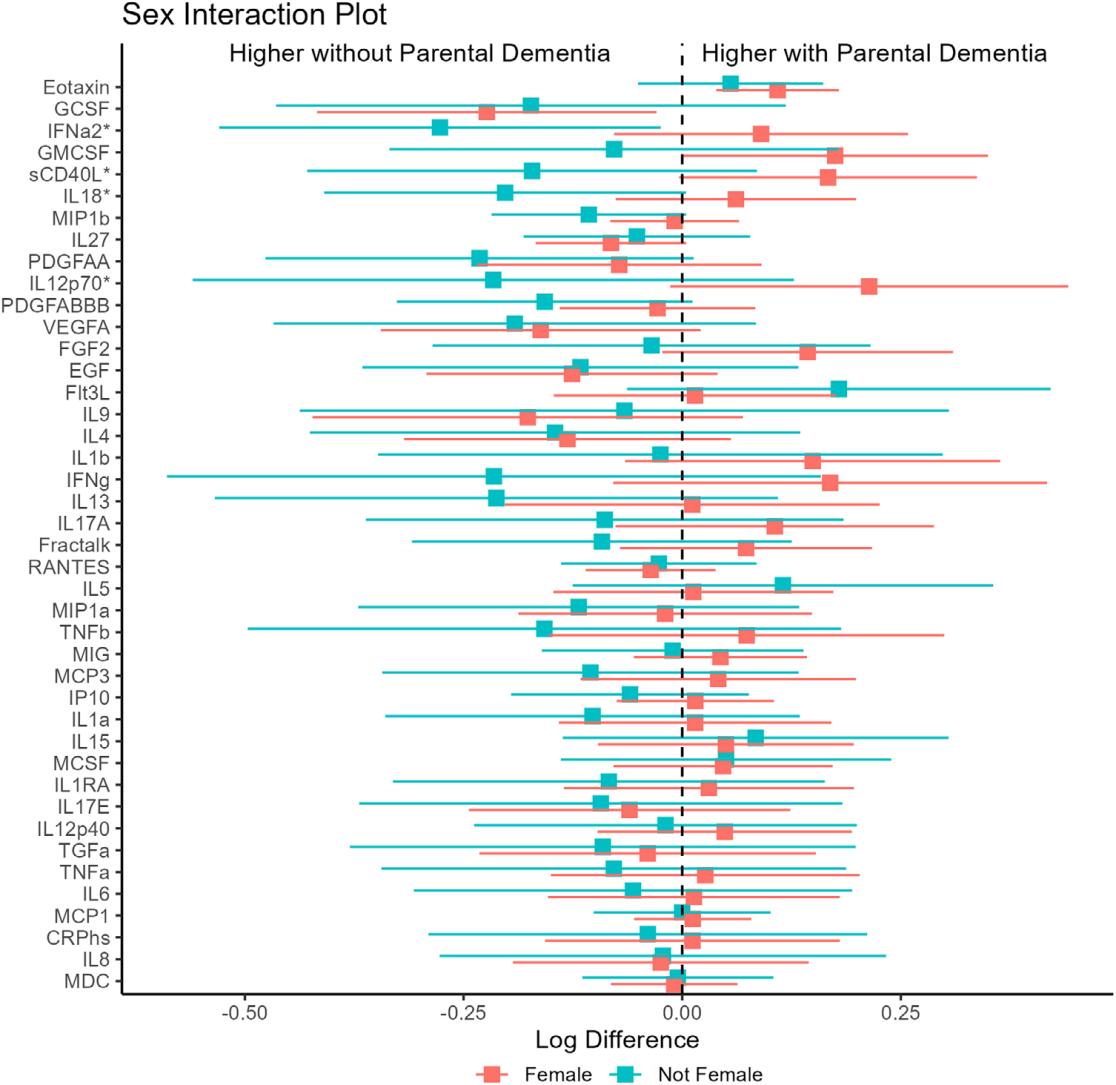
Forest plots of linear regression models examining the interactions between parental history of dementia and sex on peripheral cytokines. Beta coefficients and 95% confidence intervals are shown separately for female (red) and male (blue) participants. Asterisks (*) indicate cytokines with a significant interaction effect (*P*  <  0.05) between parental history and sex.

The association of parental dementia history with IFN‐α2 levels (β_int_ = 0.36, 95% CI: 0.06–0.66, *P* = 0.01), IL‐18 levels (β_int_ = 0.27, 95% CI: 0.02–0.51, *P* = 0.02), IL‐12p70 levels (β_int_ = 0.44, 95% CI: 0.03–0.85, *P* = 0.03), and sCD40L levels (β_int_ = 0.64, 95% CI: 0.03–0.64, *P* = 0.02) was greater among women compared to men (Figure 4).

### 3.6 Interactions between parental dementia history and *APOE* ε4 status on peripheral cytokines

*APOE* ε4 is a well‐known genetic risk factor for AD. Given this, we examined where *APOE* ε4 status modified the association between parental dementia history and peripheral cytokine levels. However, there were no interactions between *APOE* ε4 status and parental dementia history on peripheral cytokine levels (Figure S2 in supporting information).

### 3.7 Associations between psychological factors and parental dementia history

There were no differences in anxiety or depression scores between individuals with and without parental dementia history (Table S2 in supporting information).

### 3.8 Peripheral cytokines and global cognition

In all participants in the unadjusted model, EGF, eotaxin, G‐CSF, GM‐CSF, IL‐27, IL‐4, IL‐9, and VEGF‐A were significantly associated with general cognition. After adjusting for covariates, EGF, eotaxin, G‐CSF, GM‐CSF, IL‐27, IL‐4, IL‐9, and VEGF‐A remained significantly associated with general cognition (Table S3 in supporting information). There were no interactions between global cognition and parental dementia history on peripheral cytokine levels (Table 2).

**TABLE 2:** Interactions between parental dementia history and race/ethnicity on peripheral cytokines.

Cytokine	β (95% CI)	Unadj. *p* value	Adj. *p* value
EGF	−0.14 (−0.31, 0.04)	0.13	0.4
Eotaxin	0.05 (−0.03, 0.12)	0.2	0.5
G‐CSF	−0.07 (−0.28, 0.13)	0.5	0.8
GMCSF	−0.17 (−0.35, 0.01)	0.065	0.3
IL‐27	−0.02 (−0.11, 0.07)	0.6	0.8
IL‐4	−0.06 (−0.26, 0.14)	0.6	0.8
IL‐9	−0.26 (−0.51, 0)	0.048	0.3
PDGF‐AA	−0.03 (0.2, 0.15)	0.8	0.9
PDGF‐ABBB	0 (−0.12, 0.12)	>0.9	>0.9
VEGF‐A	0.06 (−0.14, 0.25)	0.6	0.8

Abbreviations: CI, confidence interval; EGF, epidermal growth factor; G‐CSF, granulocyte colony‐stimulating factor; GM‐CSF, granulocyte‐macrophage colony‐stimulating factor; IL, interleukin; PDGF, platelet‐derived growth factor; VEGF‐A, vascular endothelial growth factor A.

## 4 DISCUSSION

We examined associations between a panel of 42 peripheral inflammatory cytokines and chemokines and parental history of dementia among middle‐aged adults. We demonstrated that after adjusting for age and sex, individuals with a parental history of dementia had increased levels of the pro‐inflammatory cytokine eotaxin‐1 and decreased levels of G‐CSF, VEGF‐A, and IL‐27, although these associations did not remain statistically reliable after correction for multiple comparisons. We also found that age strengthened the association of parental history of dementia with IL‐18 and MDC. Black participants showed stronger associations of parental history of dementia with EGF and IL‐18 compared to Hispanic participants, and women with a parent AD history had higher IFN‐α2, IL‐18, IL‐12p70, and sCD40L levels than men with parental dementia history. Peripheral cytokines were associated with cognition among people without a parental history of dementia.

Eotaxin‐1/CCL11 concentrations were higher in individuals with a parental history of dementia compared to individuals without. Eotaxin‐1 is a pro‐inflammatory cytokine that increases with age,^15^ and in animal models, it is associated with reduced neurogenesis^15^ and risk for AD.^37^ Because polymorphisms within the *CCL11* gene can alter eotaxin‐1 secretion,^37^ one possible mechanism for the increased dementia risk observed in individuals with a parental history of dementia may involve genetically inherited alleles that promote higher expression of inflammatory cytokines such as eotaxin‐1. We also found that G‐CSF, VEGF‐A, IL‐27, and EGF, cytokines that are linked to neuroprotection, were decreased in individuals with a parental history of dementia. For example, G‐CSF, a growth factor important in regulating hematopoiesis, neuroprotection, and neurogenesis,^38^ is decreased in patients with AD,^39^ and associated with reduced amyloid and cognition^40^ in mouse models. Low levels of EGF predict the progression from mild cognitive impairment to clinical AD.^41^ The association between parental history of dementia and these protein concentrations in our study may reflect an early loss of neuroprotective signaling and vulnerability to cognitive decline. VEGF‐A, a growth factor implicated in brain and vascular health, is also altered in AD;^42^, ^43^, ^44^ however, the direction of its associations has been inconsistent.^2^, ^42^, ^43^, ^45^, ^46^ Our observation of reduced VEGF‐A concentrations among individuals with a parental history of dementia suggests impaired vascular and neuroprotective mechanisms in this group. Although IL‐27 has not been extensively studied in AD, studies suggest it may promote neuroprotection by regulating inflammatory cytokines, oxidative stress, and apoptosis.^47^ While its effects may be context dependent, with some studies reporting pro‐inflammatory roles in certain models, IL‐27 generally supports neuronal survival and suppresses excessive neuroinflammatory signaling.^48^ The reduction of IL‐27 observed in individuals with a parental history of dementia in our study further suggests the possibility of early loss of neuroprotection in dementia risk. We also investigated the relationship between cytokine concentrations and cognition. Interestingly, while several cytokines were associated with cognition overall, significant associations with peripheral cytokines were observed only among individuals without a parental history of dementia. This finding suggests that the relationship between circulating cytokines and cognitive function varies depending on dementia risk status. Notably, several cytokines commonly elevated in AD, such as TNF‐α, IL‐6, and IL‐1β, were not altered in individuals with a parental history of dementia, suggesting that the immune pathways contributing to the increased risk of dementia may differ from those involved in normal disease progression.

IL‐18 is a pleiotropic cytokine involved in inflammasome activation and is associated with cognitive decline and AD.^49^, ^50^, ^51^ In our study, we found that IL‐18 levels increased with age among individuals with a parental history of dementia, which could potentially reflect an accelerated inflammaging profile or an increased senescence profile, as senescent T cells express high levels of IL‐18.^52^ We also found that MDC levels increased with age among individuals with a parental history of dementia, a chemokine primarily produced by macrophages and dendritic cells that plays a role in regulating inflammation and immune cell responses.^53^ MDC levels are increased in individuals with MCI and AD compared to controls,^51^ as well as in centenarians compared to middle‐aged adults.^54^ These findings suggest that MDC may play a role in age‐related immune response in individuals with a parental history of dementia, similarly to IL‐18. Additionally, the effect of parental dementia history on EGF levels was greater in Black participants compared to Hispanic participants. As EGF is a growth factor implicated in neuroprotection, this difference may reflect population‐specific variation in neuroprotective pathways. However, we also saw that the effect of parental dementia history on IL‐18 levels was greater in Black participants compared to Hispanic participants, which, as mentioned earlier, is a pro‐inflammatory cytokine. These findings highlight that both pro‐ and anti‐inflammatory processes may be upregulated in response to parental dementia risk among Black participants, suggesting the involvement of compensatory immune mechanisms that warrant further investigation.

We also observed sex‐related differences in the associations between parental history of dementia and IFN‐α2, sCD040L, and IL‐12p70 levels. IFN‐α2, a type 1 interferon, is primarily known for its role in antiviral immunity^55^, ^56^ and women generally produce higher levels of IFN‐α2 compared to men.^57^, ^58^, ^59^ sCD40L, a key regulator of immune responses through its interaction with the CD40 receptor,^60^ has also been implicated in AD.^61^ It is encoded by the X‐linked *CD40LG* gene,^62^ and increased expression in women, potentially due to biallelic expression or X‐chromosome escape, is associated with heightened autoimmune risk.^63^ Our findings may reflect a similar mechanism in which women with parental dementia history are predisposed to increased inflammation via CD40L‐related immune signaling. We also observed a stronger relationship between parental dementia history and IL‐12p70 levels in women. IL‐12 is elevated in the brain and CSF of people with AD and MCI,^64^ and deletion of IL‐12 ameliorates AD pathology in mice.^65^ While sex differences in IL‐12 have not been reported in AD, experimental studies show that after vaccination, CD8^+^ T cells in female mice become more terminally differentiated in the presence of IL‐12.^66^ Together, these findings suggest that elevated levels of IL‐12 may reflect an accelerated immune aging or senescent profile in women by promoting terminal differentiation of CD8^+^ T cells, potentially contributing to sex‐specific differences in AD risk in the context of parental history of dementia.

Many of the cytokines implicated in this study, such as IL‐18, IL‐12, MDC, and CD40L, play critical roles in regulating the adaptive immune system, particularly T cell activation, differentiation, and communication with antigen‐presenting cells.^52^, ^67^, ^68^, ^69^ Given the bidirectional relationship between cytokine signaling and immune cell function, these altered cytokine levels may reflect underlying changes in immune cell composition or responsiveness in individuals with a parental history of dementia risk. Future studies should investigate whether parental history of dementia is associated with shifts in adaptive immune cell subsets, such as effector memory T cells or regulatory populations, which could help clarify the cellular mechanisms underlying early immune changes in at‐risk populations and their contribution to dementia risk.

Our study has several limitations worth noting. First, its cross‐sectional design prevents us from determining whether altered peripheral inflammation is a cause or consequence of dementia risk in individuals with a parental history. Longitudinal studies are needed to assess temporal relationships and clinical outcomes. Our study sample was drawn exclusively from individuals residing in the New York City metro area, which may limit the generalizability of our findings to broader populations. Third, most associations did not remain statistically reliable after correction for multiple comparisons and should therefore be considered hypothesis generating and confirmed in larger, independent samples. We were also unable to assess potential mediators, such as caregiver burden, which may contribute to psychological stress and influence peripheral inflammation. However, we did not observe differences in anxiety or depressive symptoms by parental dementia status, suggesting that mood‐related factors are unlikely to account for the observed inflammatory differences. Finally, although we observed several interaction effects, our sample size, particularly within stratified subgroups, may limit the precision and generalizability of these estimates, and replication in larger cohorts is warranted.

In conclusion, we found a pro‐inflammatory profile among individuals with a parental history of dementia. Our findings point toward a trend that peripheral inflammation may contribute to the elevated dementia risk observed in this group, with effects by age, sex, and race and ethnicity. Many of the implicated cytokines are closely linked to adaptive immune composition and responsiveness. Notably, many of the identified cytokines are closely linked to adaptive immune function, particularly in T cells, raising the possibility that a parental history of dementia influences immune composition and responsiveness. Together, these results add to growing evidence that immune dysregulation may play an important role in the pathogenesis and intergenerational transmission of dementia risk.

## CONFLICT OF INTEREST STATEMENT

The authors declare no conflicts of interest. Author disclosures are available in the supporting information.

## CONSENT STATEMENT

All participants in the Offspring Study provided written informed consent.

## Supporting information



Supporting Information

Supporting Information

Supporting Information

Supporting Information

Supporting Information

Supporting Information
