# Supplementary material for: Associations between parental history of dementia and plasma markers of inflammation in a multi‐ethnic middle‐aged community of adults
Source: Alzheimers Dement. 2026 Apr 12;22(4):e71355. doi: 10.1002/alz.71355 (PMC13071171; doi:10.1002/alz.71355)
Supplement: Supplementary file 1 — Supporting Information [file ALZ-22-e71355-s001.docx]

**Supplementary Material**


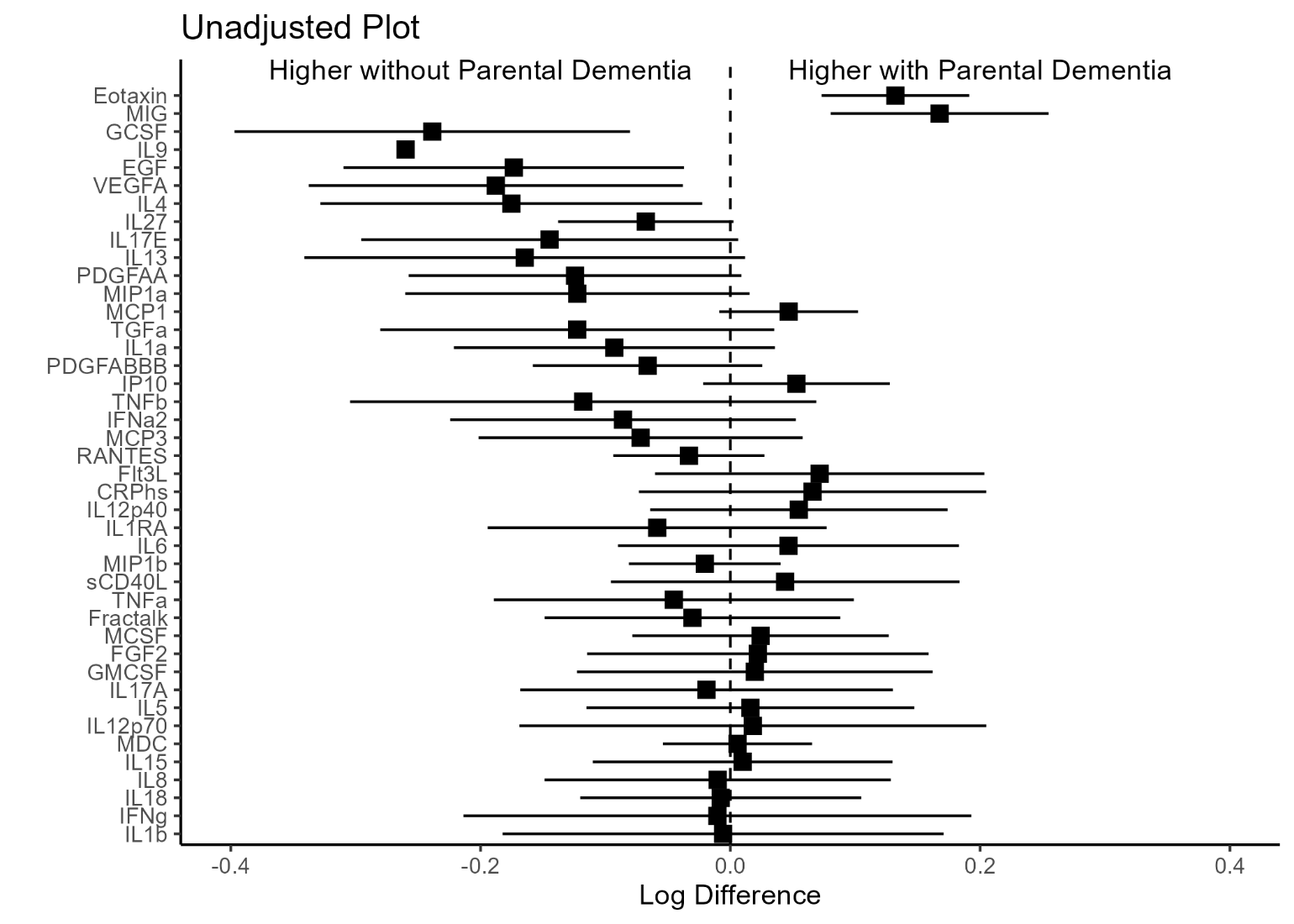


**Supplementary Figure 1**. Forest plots of unadjusted linear regression models examining the associations between peripheral cytokines and chemokines and parental history of dementia. Results are shown separately for individuals with and without a parental history of dementia. Beta coefficients reflect the difference in log-transformed cytokine concentrations between individuals with and without a parental history of dementia. Error bars represent 95% confidence interval.
