# Supplementary material for: Associations between parental history of dementia and plasma markers of inflammation in a multi‐ethnic middle‐aged community of adults
Source: Alzheimers Dement. 2026 Apr 12;22(4):e71355. doi: 10.1002/alz.71355 (PMC13071171; doi:10.1002/alz.71355)
Supplement: Supplementary file 2 — Supporting Information [file ALZ-22-e71355-s003.docx]

**Supplementary Materials**


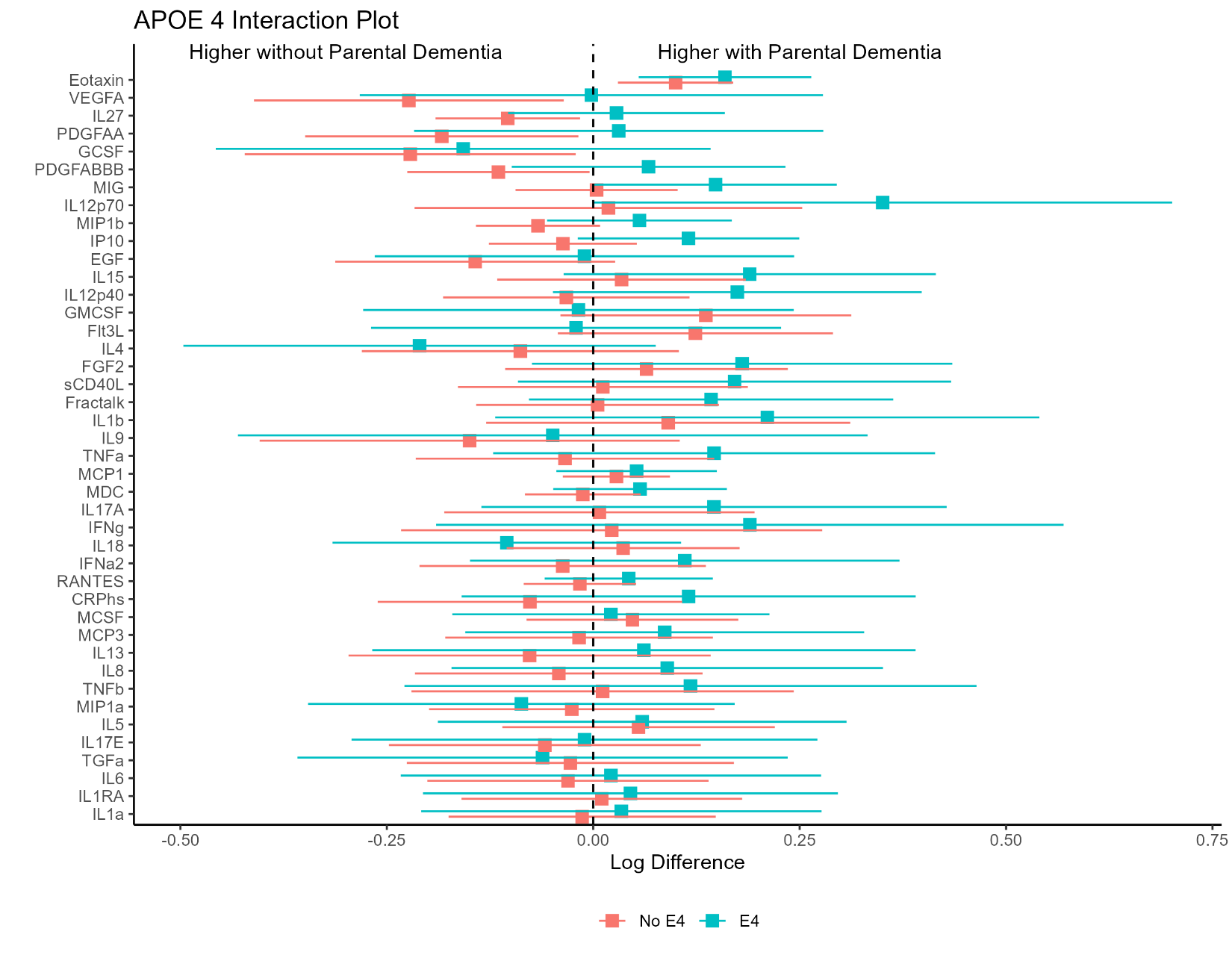


**Supplementary Figure 2**. Forest plots of linear regression models examining the interactions between parental history of dementia and APOE-ε4carrier status. Beta coefficients and 95% confidence intervals are shown separately for non-APOE-ε4 carriers (red) and APOE-ε4 carriers.
