# Supplementary material for: Associations between parental history of dementia and plasma markers of inflammation in a multi‐ethnic middle‐aged community of adults
Source: Alzheimers Dement. 2026 Apr 12;22(4):e71355. doi: 10.1002/alz.71355 (PMC13071171; doi:10.1002/alz.71355)
Supplement: Supplementary file 3 — Supporting Information [file ALZ-22-e71355-s002.docx]

| **Table S1. Dementia diagnosis across groups** | | |
| --- | --- | --- |
| **Characteristics** | No Parental AD^1^ (N=778) | Parental AD^1^ (N=446) |
| Last dementia etiology |  |  |
| Probable Alzheimer's Disease | 0 (NA%) | 284 (67%) |
| Alzheimer's with Stroke | 0 (NA%) | 47 (11%) |
| Alzheimer's w/ other concomitant disease | 0 (NA%) | 69 (16%) |
| Parkinson's disease dementia | 0 (NA%) | 7 (1.7%) |
| Vascular Dementia | 0 (NA%) | 5 (1.2%) |
| Secondary Dementia: Metabolic | 0 (NA%) | 1 (0.2%) |
| Other Dementia | 0 (NA%) | 6 (1.4%) |
| Dementia, Cause Unknown | 0 (NA%) | 4 (0.5%) |
| Dementia with Lewy Body | 0 (NA%) | 3 (0.7%) |
| *^1^*n (%) |  |  |
