# Supplementary material for: Associations between parental history of dementia and plasma markers of inflammation in a multi‐ethnic middle‐aged community of adults
Source: Alzheimers Dement. 2026 Apr 12;22(4):e71355. doi: 10.1002/alz.71355 (PMC13071171; doi:10.1002/alz.71355)
Supplement: Supplementary file 4 — Supporting Information [file ALZ-22-e71355-s006.docx]

| **Table S2.** ***Associations between parental dementia history and psychological factors*** | | | |
| --- | --- | --- | --- |
| **Characteristic** | **No Parental Dementia** N = 781*^1^* | **Parental Dementia** N = 426*^1^* | **p-value***^2^* |
| Anxiety | 2.3, 2.7; (0.0-14.0) | 2.2, 3.0; (0.0-14.0) | 0.8 |
| Unknown | 58 | 31 |  |
| Depression | 6.8, 5.8; (0.0-30.0) | 6.1, 5.8; (0.0-27.0) | 0.063 |
| Unknown | 75 | 60 |  |
| *^1^*Mean, SD; (Min-Max) | | | |
| *^2^*One-way analysis of means (not assuming equal variances) | | | |

Note: SD= Standard deviation
