# Supplementary material for: Associations between parental history of dementia and plasma markers of inflammation in a multi‐ethnic middle‐aged community of adults
Source: Alzheimers Dement. 2026 Apr 12;22(4):e71355. doi: 10.1002/alz.71355 (PMC13071171; doi:10.1002/alz.71355)
Supplement: Supplementary file 5 — Supporting Information [file ALZ-22-e71355-s004.docx]

| **Table S3.** ***Associations between peripheral cytokines and general cognition*** | | | | | | |
| --- | --- | --- | --- | --- | --- | --- |
| **Unadjusted** | | | | **Adjusted** | | |
| **Cytokine** | β (95% CI) | Unadj. p-value | Adj. p-value | β (95% CI) | Unadj. p-value | Adj. p-value |
| EGF | .17 (.1, .24) | <0.001 | <0.001 | .16 (.08, .24) | <0.001 | <0.001 |
| Eotaxin | -.09 (-.12, -.06) | <0.001 | <0.001 | -0.05 (-.09, -.02) | 0.002 | 0.003 |
| G-CSF | .15 (.07, .23) | <0.001 | <0.001 | 0.16 (.07, .26) | <0.001 | 0.002 |
| GMCSF | .13 (.06, .21) | <0.001 | <0.001 | 0.1 (.01, .18) | 0.024 | 0.031 |
| IL-27 | .06 (.02, .09) | 0.001 | 0.002 | .08 (.04, .12) | <0.001 | <0.001 |
| IL-4 | .13 (.05, .21) | <0.001 | 0.002 | .14 (.05, 0.23) | 0.003 | 0.004 |
| IL-9 | .23 (.13, .33) | <0.001 | <0.001 | .17 (.05, .28) | 0.005 | 0.007 |
| PDGFAA | .05 (-.02, .11) | 0.2 | 0.2 | .06 (-.02, .14) | 0.11 | 0.12 |
| PDGFABBB | .02 (-.02, .07) | 0.3 | 0.3 | .04 (-.01, 0.1) | 0.12 | 0.12 |
| VEGF-A | .11 (.03, .19) | 0.005 | 0.006 | .14 (.06, 0.23) | 0.001 | 0.003 |

Note: CI= Confidence Interval
